# Different types of diabetes mellitus and risk of thyroid cancer: A meta-analysis of cohort studies

**DOI:** 10.3389/fendo.2022.971213

**Published:** 2022-09-23

**Authors:** Wen-wu Dong, Da-Lin Zhang, Zhi-Hong Wang, Cheng-Zhou Lv, Ping Zhang, Hao Zhang

**Affiliations:** Department of Thyroid Surgery, The First Hospital of China Medical University, Shenyang, China

**Keywords:** diabetes mellitus, thyroid cancer, meta-analysis, cohort study, risk

## Abstract

**Objective:**

Sex-specific thyroid cancer risk exists in patients diagnosed with diabetes mellitus (DM). However, thyroid cancer risk in different types of DM is still unclear. This meta-analysis aims to identify the real correlation between different types of DM and thyroid cancer risk in both sexes.

**Methods:**

Studies were identified by an electronic search of PubMed, EMBASE, and Cochrane Library on 16 January 2022. A random-effects model was used to estimate the relative risks (RRs). The Cochran’s Q and I^2^ statistics were computed to detect heterogeneity between studies.

**Results:**

In comparison with non-DM counterparts, patients with DM had a 1.32-fold higher risk of thyroid cancer (95% CI, 1.22–1.44) with 1.26-fold (95% CI, 1.12–1.41) in men and 1.36-fold (95% CI, 1.22–1.52) in women, respectively. Subgroup analysis by the type of DM showed that the RR of thyroid cancer in patients with type 2 diabetes was 1.34 (95% CI, 1.17–1.53) in the study population with 1.32 (95% CI, 1.12–1.54) in men and 1.37 (95% CI, 1.12–1.68) in women, respectively; the RR of thyroid cancer was 1.30 (95% CI, 1.17–1.43) in patients with gestational diabetes; the risk of thyroid cancer in patients with type 1 diabetes was 1.51-fold in women but not in men. Although there were some heterogeneities, it did not affect the above results of this study.

**Conclusion:**

This study indicates that, compared with non-DM individuals, patients with any type of DM have an elevated thyroid cancer risk. This positive correlation between type 2 diabetes and thyroid cancer risk exists in both men and women.

**Systematic Review Registration:**

https://www.crd.york.ac.uk/prospero/, CRD42022304028.

## Introduction

The incidence of thyroid cancer has increased greatly in the past 3 decades (1).Thyroid cancer is the most popular malignancy in endocrine system, accounting for an estimated 43,800 new cases with threefold higher overall incidence rates in American women in 2022 (2). On the basis of the latest Chinese cancer statistics, thyroid cancer is the fourth most frequent cancer in women with its incidence rising by 12.4% every year (3, 4). The rapidly increased incidence of thyroid cancer is considered to be predominantly owing to overdiagnosis. However, it is not yet clear whether the epidemic of thyroid cancer is also caused by exposure to certain risk factors (5).

Risk factors of thyroid cancer have not yet been established. Ionizing radiation and family history of thyroid cancer are the accepted risk factors of thyroid cancer. Additional potential risk factors include obesity and reproductive and environmental factors (6–8). The potential impact of other risk factors for thyroid carcinogenesis warrants further exploration. Here, we explore the possible impact of diabetes mellitus (DM) on subsequent thyroid cancer.

DM represents one of the most rapidly increasing global public health problems. The worldwide prevalence of DM is estimated to grow from 2.8% to 4.4% between 2000 and 2030 and projected to be one of the five leading contributors to disease burden by 2030 (9). China has the largest number of DM people in the world with the rise in prevalence from 0.9% in 1980 to 10.9% in 2013 and more than 90% of whom have type 2 diabetes (T2D) (10, 11). DM has been positively associated with the risk of cancer at different sites, including prostate, oral, breast, and pancreas (12–15). The prevalence of both DM and thyroid cancer has increased all over the world, providing theoretic support that DM may be a driver for thyroid cancer, although early studies did not find an association between them (16). The insufficient cancer cases among the exposed group might account for the lack of association. Furthermore, all of these studies were launched in the USA, possibly leading to the insufficiency of the heterogeneity of exposure and the power of statistics. Two recent meta-analyses have illustrated that the risk of thyroid cancer in female patients with DM has significantly increased in comparison with their non-diabetic counterparts (17, 18). However, there are still some shortcomings in these studies. First, the type of DM was not differentiated and thyroid cancer risk caused by various kinds of DM could not be highlighted. Second, the previous meta-analysis included patients with metabolic syndrome, which may lead to confounding bias. Furthermore, the correlation between DM and thyroid cancer in male patients is not clear, owing to the small sample size. Thus, this meta-analysis was designed to determine the association between various kinds of DM and the risk of thyroid cancer and whether the discrepancy exists in sex based on the available cohort studies.

## Methods

This study was designed and carried out according to the guidelines for the Preferred Reporting Items for Systematic Reviews and Meta-Analyses (PRISMA 2020) (19). The protocol has been registered in the International Prospective Register of Systematic Reviews platform. The registration number of this meta-analysis is CRD42022304028.

### Search strategy

A literature search-up was conducted using Cochrane Library, EMBASE, and PubMed and without language restrictions from databases on 16 January 2022. Medical Subject Heading (MESH) terms and keywords were (“Diabetes Mellitus” OR “Diabetes”) AND (“Thyroid Tumor” OR “Thyroid Neoplasm*” OR “Thyroid Adenoma*” OR “Thyroid Cancer*” OR “Thyroid Carcinoma*”). Furthermore, a manual screening of the reference lists of selected studies was also conducted to find extra potentially relevant studies. The integrated search strategy is shown in the **Appendix (Table 1–3)**.

### Eligibility criteria

Studies were included if the following criteria were met (1): cohort studies, whether prospective or retrospective (2); the exposed group could be patients with any type of DM, the most common being T2D, T1D, or gestational diabetes (GD), and the control group consisted of non-DM individuals (3); thyroid cancer risk as the outcome that expressed as a hazard ratio (HR), adjusted odds ratio (OR), relative risk (RR), or standardized incidence ratio (SIR); (4) thyroid cancer should be a risk occurring naturally under observation, and drug intervention to reduce thyroid cancer risk was not considered.

### Exclusion criteria

The exclusion criteria were (1) conference abstracts or erratum, (2) duplicate published studies based on the same observation population, and (3) incomplete data or no interested outcome.

### Research selection

The eligible studies were independently screened by two investigators (Wenwu Dong and Dalin Zhang). First, they excluded duplicate and irrelevant literatures by title and abstract. Then, each of them independently read the full text of each potentially eligible article and finally identified all studies. Any disagreement was resolved by discussion with a third investigator (Hao Zhang) until consensus was achieved.

### Data extraction

Two investigators (Wenwu Dong and Dalin Zhang) independently extracted data. The following pertinent information was included: name of first author, country, publication year, sample size, DM type, cases of thyroid cancer, mean age at baseline, follow-up years, diagnosis criteria of thyroid cancer, and adjusted confounders (20).

### Risk-of-bias assessment

The quality of the selected studies was assessed on the basis of the Newcastle-Ottawa Quality Assessment Scale (NOS). The range of scores was from 0 to 9, and the higher the score, the higher the study quality. NOS scores ≥ 7, 4–6, and 0–3 mean high, medium, and low quality, respectively.

### Statistical analysis

SIR, RR, HR, or adjusted OR and 95% confidence intervals (CI) were extracted to estimate thyroid cancer risk in different types of DM. Because of the low attack rate of thyroid cancer, RR is approximately equal to OR, HR, and SIR, so a pooled analysis can be performed. Heterogeneity was assessed using the chi-square test and I^2^ value. *P*-value < 0.1 or I^2^ > 50% was considered to indicate significant heterogeneity, and random-effects model was adopted. Otherwise, a fixed-effects model was employed. The sensitivity analysis was conducted to examine the robustness of our results. Subgroup analysis was conducted for different types of DM in different sex. Finally, funnel plots and Egger’s regression test were conducted to assess publication bias. A two-sided *P*-value less than 0.05 was considered significant. Statistical analyses were performed using the Stata software (version 15.1).

## Results

### Literature search

**Figure 1:**
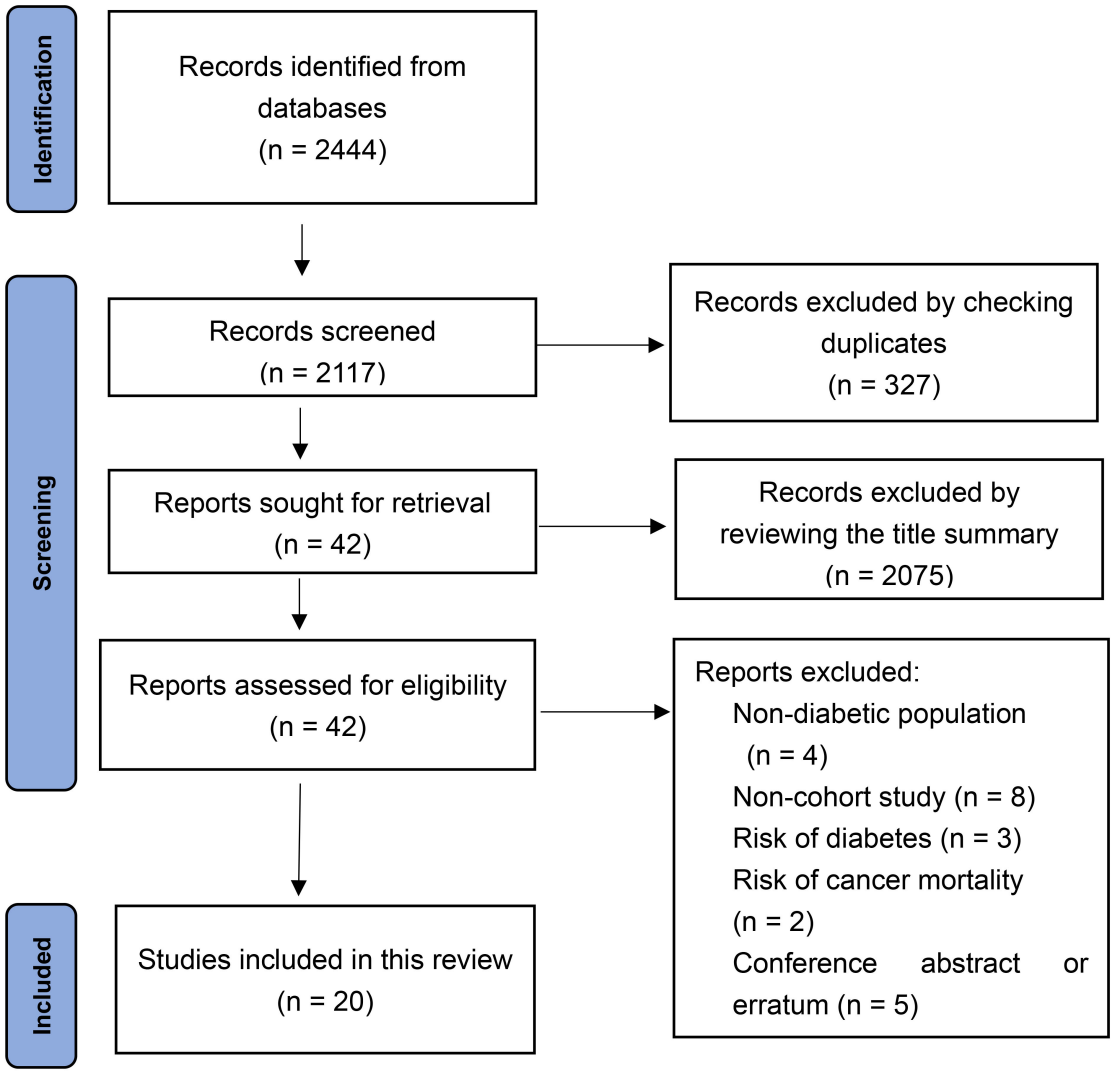
Flowchart of study screening.

From the electronic search, a total of 2,444 records were identified. A total of 327 articles were excluded by checking duplicates. Another 2,075 articles were excluded after reviewing the title summary. The 42 remaining articles were examined in full text. Finally, 20 cohort studies were included according to the inclusion criteria (16, 21–39). The literature search algorithm is shown in **Figure 1**.

### Study characteristics

The included cohort studies (16, 21–39) were published between 1991 and 2019 from nine countries and regions, including Europe, Asia, and America, with the largest number of studies from Asia. Types of DM varied in these cohort studies. T2D (21, 24–26, 36–38) was the most frequently studied, followed by GD (22, 23, 27, 28). Three studies (34, 35, 39) did not identify the type of DM, and only one (29) reported thyroid cancer risk in patients with T1D. The sample size of the included studies was greater than 300,000, and a total of 11,091 cases of thyroid cancer occurred during follow-up. The mean follow-up time ranged from 3.0 to 20.8 years. Most studies on the diagnosis criteria of thyroid cancer were in accordance with International Classification of Diseases (ICD), and the confounding factors (e.g., sex and body mass index) were well controlled. The baseline characteristics of the included studies are presented in **Table 1**.

**Table 1:** Basic characteristics of the included cohorts.

Author	Year	Country	Diabetes type	Sample	Thyroid cancer cases	Mean ageat baseline	Mean follow-up years	Diagnosis of thyroid cancer	Confounders adjusted	NOS scores
Linkeviciute (1)	2019	Lithuania	T2D	127,290	232	62.4	6.5	ICD-10	NR	6
Pace (2)	2019	Canada	GD	68,588	221	≤30	13.1	ICD-10	Race, BMI, maternal age at delivery, year of delivery, and ethnicity	7
Qi (3)	2019	China	T2D	410,191	510	61.8	3	ICD-10	Sex and age	7
Saarela (4)	2018	Finland	T2D	428,326	600	≥30	13	ICD-10	NR	6
Peng (5)	2019	China	GD	990,572	1,514	31.61/28.83	13	ICD-9	Age, infertility and kidney disease, dyslipidemia, liver disease, and hypertension	8
Fang (6)	2018	China	T2D	51,324	141	60	10	ICD-10	NR	7
Han (7)	2018	Korea	GD	102,900	1,953	28.25/27.28	10	ICD-10	Smoking, maternal age, FBG, and BMI before pregnancy	8
Carstensen (8)	2016	Denmark	T1D	3.9 million person-years	NR	≤40	20.8	ICD-10/ ICD-7	NR	6
Luo (9)	2016	USA	T2D	147,934	391	63.2/63	15.9	NR	Age, ethnicity, education, smoking status, recreational physical activity, alcohol intake, history of hormone therapy use, and previous thyroid disease	7
Dankner (10)	2016	Israel	T2D	1,152,122	833	21–89	11	ICD-Oncology	Age, ethnicity, and socioeconomic status	8
Bejaimal (11)	2016	Canada	GD	149,049	632	32	10	NR	Number of physician visits in 3 years before the index date and income	6
Xu (12)	2015	China	T2D	36,379	29	58.44/59.37	3.78	ICD-10	Age and sex	7
Lo (13)	2012	China	T2D	1,790,868	1,309	60.5/60.4	3.5	ICD-9	Age, sex, urbanization, hypertension, and hyperlipidemia	8
Kitahara (14)	2012	USA	T2D	674,491	818	60	10.5 (median)	ICD-Oncology	Race, education, sex, smoking, marital status, alcohol intake, BMI, cigarette, and cohort	8
Aschebrook-Kilfoy (15)	2011	USA	Untyped diabetes	496,548	525	62	10	ICD-Oncology	Age, sex, smoking status, education, BMI, family history of cancer, and race/ethnicity	7
Atchison (16)	2010	USA	Untyped diabetes	4,501,578	1,053	57.5/51.5	10.5–11.9	ICDA-8/ICD-9	Race, Age, latency, time, and alcohol-related conditions, number of visits, COPD, and obesity	8
Johnson (17)	2011	Canada	T2D	370,200	126	61	4.3	ICD-9	Sex, birth year, and index year	6
Chodick (18)	2010	Israel	T2D	100,595	114	47	8	ICD-9	Age, history of cardiovascular diseases, region, BMI, and SES level	7
Adami (19)	1991	Sweden	Untyped diabetes	51,008	19	≥20	5.2	ICD-7	Age and sex	6
Hemminki (20)	2010	Sweden	T2D	125,126	71	≥39	5	ICD-7~10	Obesity	7

ICD, International Classification of Diseases; T2D, type 2 diabetes; T1D, type 1 diabetes; GD, gestational diabetes; NR, no report; FBG, fasting blood glucose; BMI, body mass index; COPD, chronic obstructive pulmonary disease.

### Quality assessment

Specific assessments with the NOS scores are shown in **Table 1**. Six studies with a score of 6 were deemed of moderate quality, and 14 studies with a score of ≥ 7 were classified as high quality. The mean score was seven points, suggesting a high overall quality.

### Sex-specific risk of thyroid cancer in any type of DM

**Figure 2:**
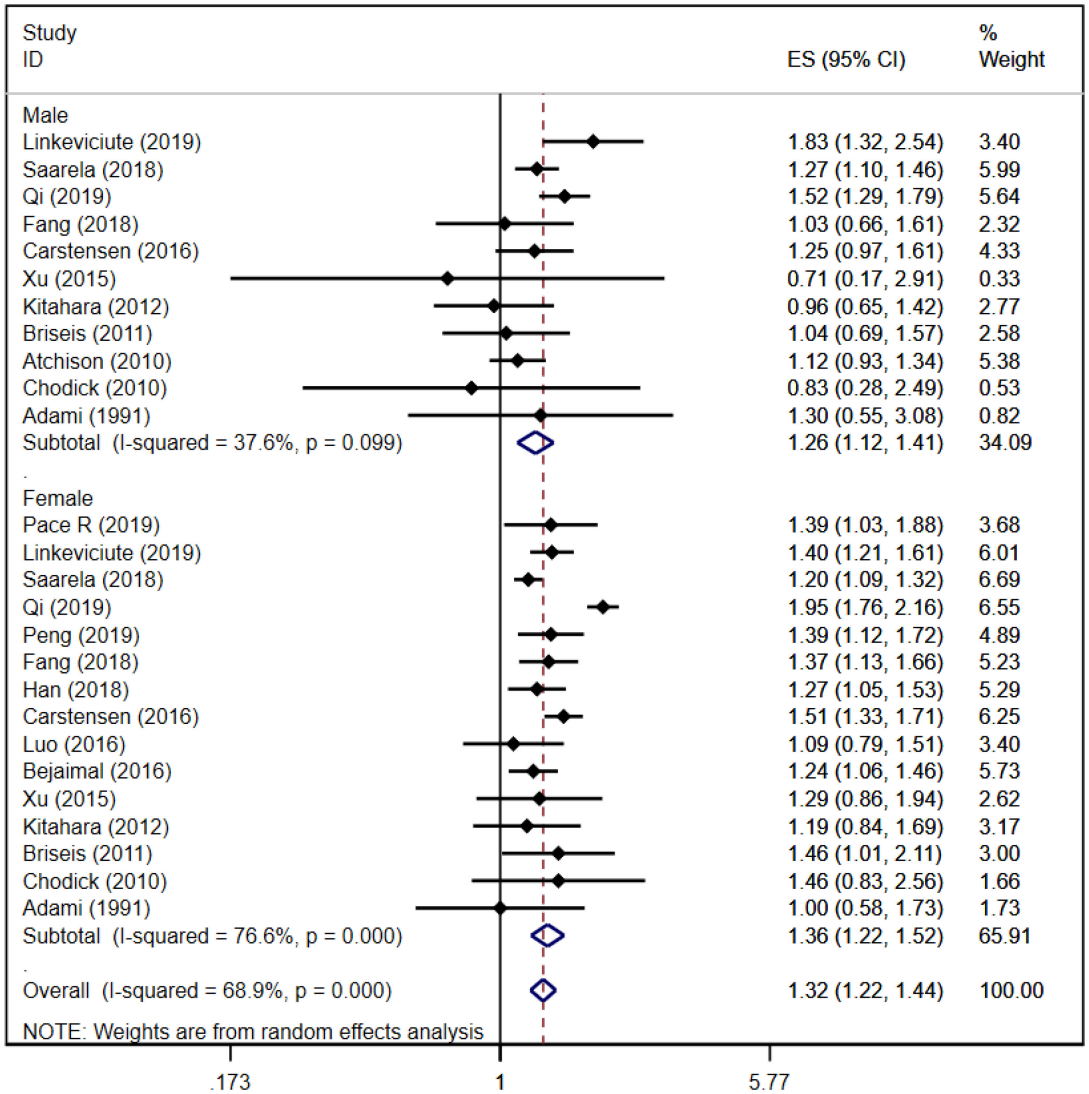
Forest plots of sex-specific thyroid cancer risk in any type of diabetes.

A total of 11 (16, 21, 24–26, 29, 32, 34, 35, 37, 39) and 15 (16, 21–29, 32, 34, 35, 37, 39) cohort studies reported that thyroid cancer risk in male and female patients with DM, respectively. The pooled analysis showed that thyroid cancer risk in male patients was [RR = 1.26, 95% CI (1.12, 1.41), I^2^ = 36.7%, *P* = 0.000]; thyroid cancer risk in female patients was [RR = 1.36, 95% CI (1.22, 1.52), I^2^ = 76.6%, *P* = 0.000]; and the total thyroid cancer risk in the overall study populations was [RR = 1.32, 95% CI (1.22, 1.44), I^2^ = 68.9%, *P* = 0.000]. The risk of thyroid cancer in female patients was slightly higher than that in male patients. Forest plots of sex-specific thyroid cancer risk in any type of DM is shown in **Figure 2**. Because of some heterogeneity, sensitivity analyses were performed by sequentially excluding each study in the pooled analysis. The results did not affect the overall conclusions, suggesting that the conclusions of this study are reliable. Sensitivity analysis plot is in **Appendix Figure 1**.

### Risk of thyroid cancer in T2D

**Figure 3:**
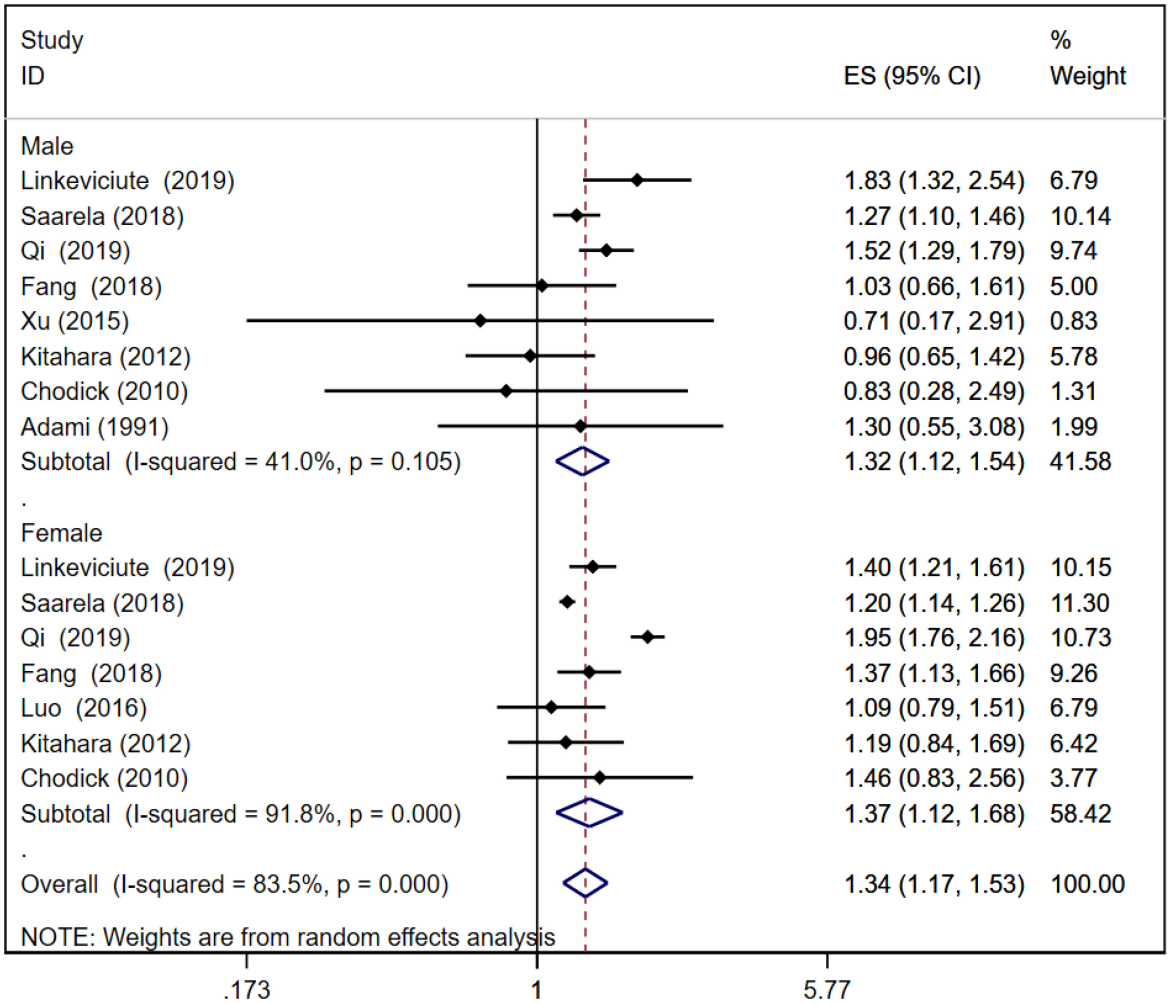
Forest plots of thyroid cancer risk in type 2 diabetes.

A total of eight (16, 21, 24–26, 32, 37, 39) and seven (16, 21, 24–26, 31, 37) cohort studies reported thyroid cancer risk in male and female patients with T2D, respectively. The pooled analysis showed that thyroid cancer risk in male patients was [RR = 1.32, 95% CI (1.12, 1.54), I^2^ = 41.0%, *P* = 0.001]; thyroid cancer risk in female patients was [RR = 1.37, 95% CI (1.12, 1.68), I^2^ = 91.8%, *P* = 0.002]; and the total thyroid cancer risk in T2D patients was [RR = 1.34, 95% CI (1.17, 1.53), I^2^ = 83.5%, *P* = 0.000]. In T2D, thyroid cancer risk in female patients was slightly higher than that in male patients. Forest plots of thyroid cancer risk in T2D is shown in **Figure 3**. Because of large heterogeneity, sensitivity analyses were performed by excluding each study sequentially from the pooled analysis, but the overall conclusion was not affected, suggesting that the results of our meta-analysis are robust. Sensitivity analysis plot is presented in **Appendix Figure 2**.

### Risk of thyroid cancer in GD

**Figure 4:**
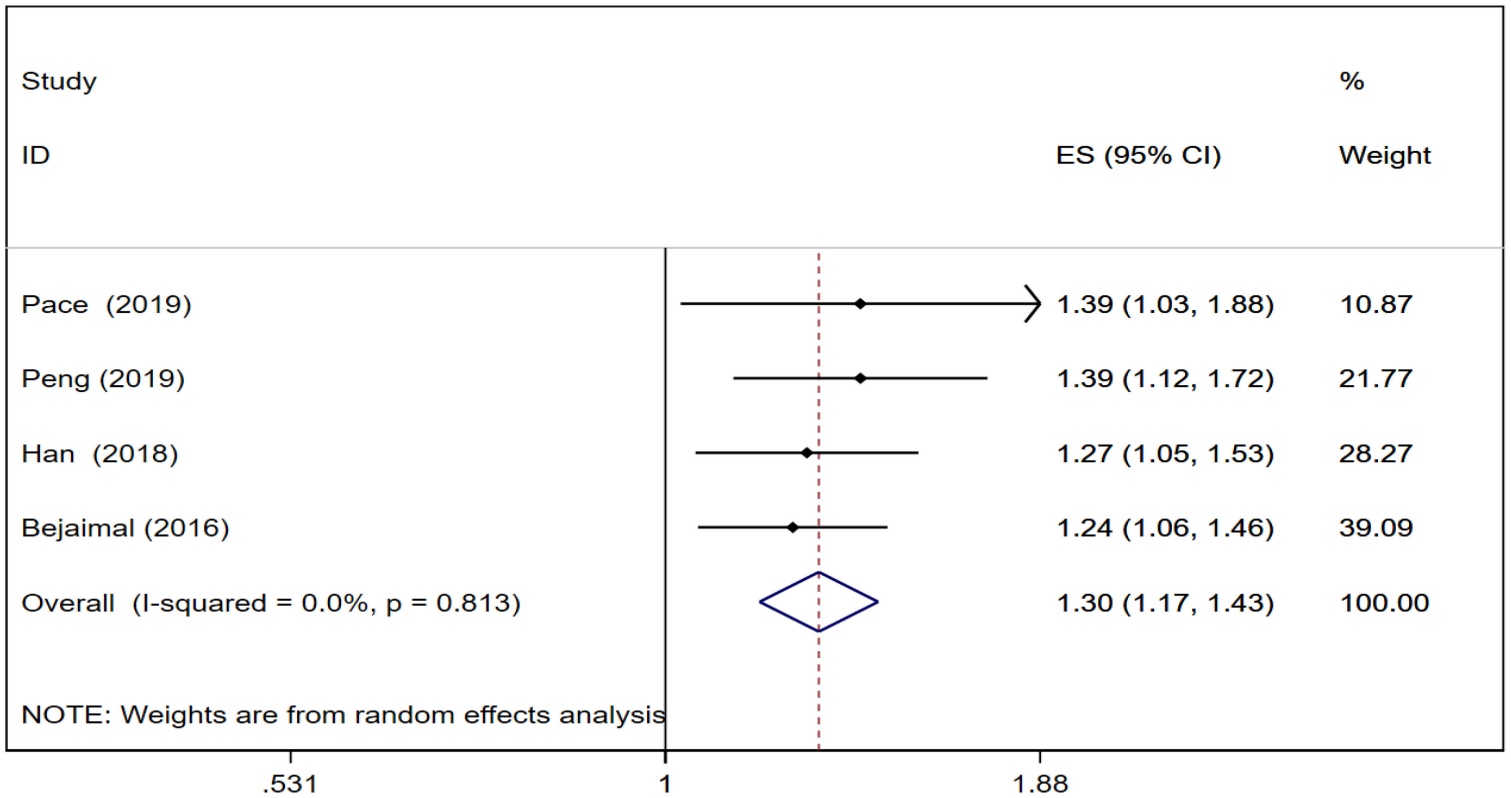
Forest plots of thyroid cancer risk in gestational diabetes.

Four cohort studies (22, 23, 27, 28) reported thyroid cancer risk in GD. The pooled analysis showed that thyroid cancer risk was [RR = 1.30, 95% CI (1.17, 1.43), I^2^ = 0.0%, *P* = 0.000]. Forest plots of thyroid cancer risk in GD is shown in **Figure 4**.

### Risk of thyroid cancer in T1D

Only one cohort study (29) reported that the risk of thyroid cancer increased in patients with T1D. Among them, no association was found between male patients and the risk of thyroid cancer. The risk of thyroid cancer in female patients was 1.51-fold.

### Publication bias

**Figure 5:**
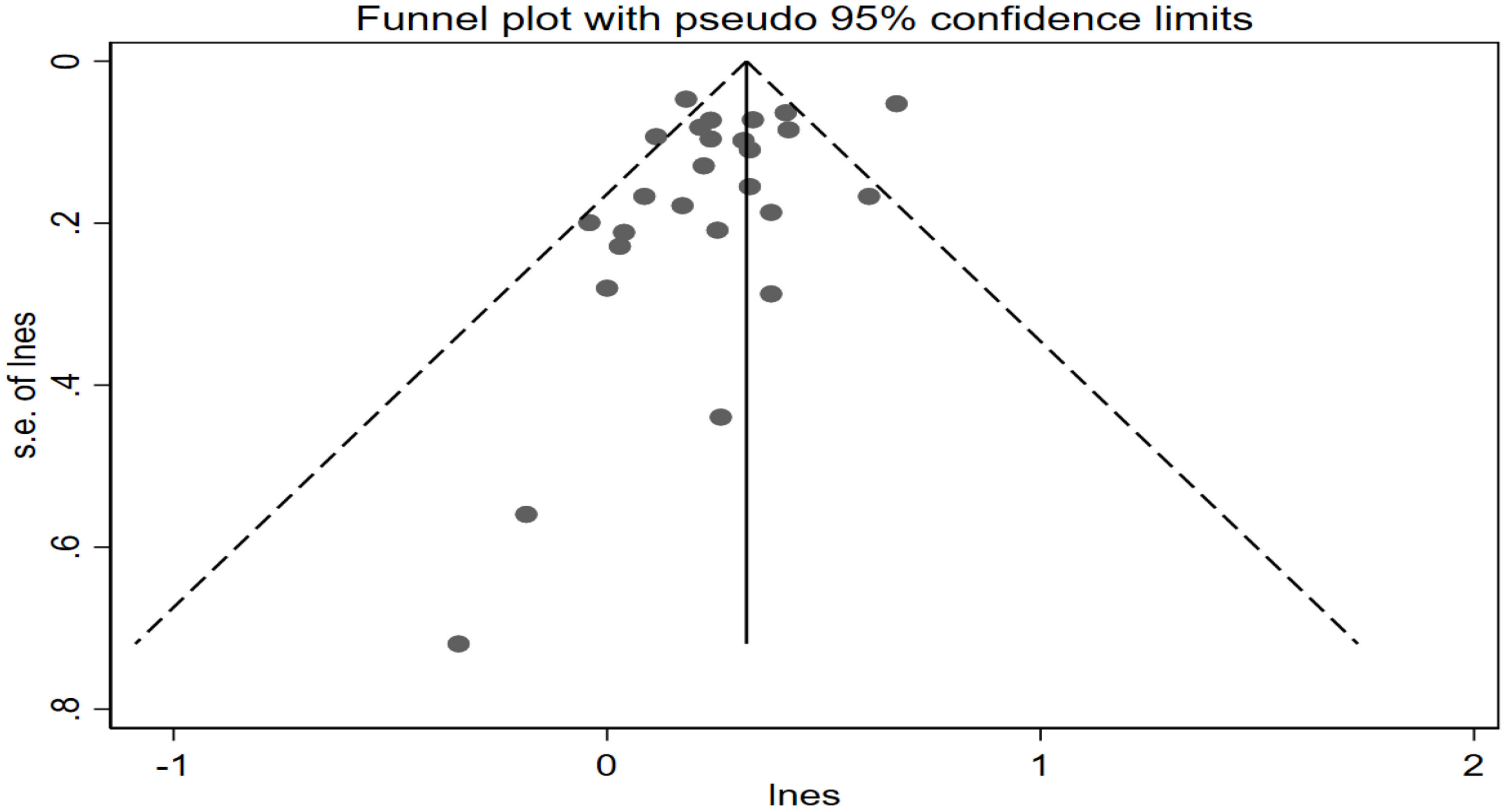
Funnel plot of risk of thyroid cancer in diabetes.

No evidence of publication bias was observed in the visual distribution of the funnel plot (**Figure 5**). The result of Egger’s regression tests is *P* = 0.108 ≥ 0.05, indicating no publication bias in this meta-analysis.

## Discussion

This meta-analysis of 20 cohort studies included more than 300,000 individuals, providing evidence that thyroid cancer risk increased approximately 30% in DM for the entire study populations, with a 36% increase among female patients and a 26% increase among male patients. The increased thyroid cancer risk varies among patients with different kinds of DM with 34%, 30%, and 51% in patients with T1D, GD, and T2D, respectively. Unexpectedly, the correlation between T2D and thyroid cancer risk existed not only in female patients but also in male patients. This contradicts previous reports that speculated that this correlation between DM and thyroid cancer was prominent in female patients but not in male patients (17, 18). Our positive findings in men were mainly due to the more accurate patient enrolment, the subgroup analysis of different types of DM and the enlargement of sample size.

The global prevalence of DM in 2017 was 8.8% with a further increase expected to 9.9% by the year 2045, nearly 90% of which was T2D (40). Our findings indicated that T2D was a risk factor for thyroid cancer in both male and female patients, and therefore, male sex was not a protective factor for thyroid cancer in patients with T2D. However, the association between T1D and the risk of thyroid cancer was evident in female patients but not in male patients reported by only one cohort study (29). Thus, more research needs to be undertaken to confirm the findings and to explore the underlying molecular mechanisms. GD was also a highly prevalent condition affecting 9.3%–25.5% of pregnant women (41). Thus, pregnant women were potential susceptible population of thyroid cancer. Understanding potential sex discrepancy in risk of thyroid cancer is critical from both a population health and clinical perspective. From the perspective of public health, assessment of sex differences sends messages to targeted public health and is conducive to draw projections of the future disease burden of thyroid cancer and estimating relevant public health costs. Clinically, knowledge of sex differences may contribute to patient selection for thyroid cancer screening.

Some molecular biological mechanisms may explain this correlation. First, chronic elevated insulin levels observed in patients with DM may influence thyroid cancer risk, which was mediated by insulin receptors overexpressed in cancer cells and tissues (42). Insulin may activate insulin and insulin-like growth factor-1 (IGF-1) pathway to inhibit cell apoptosis and promote proliferation. Insulin may also activate mitogen-activated protein kinase (MAPK) and the phosphatidylinositide 3-kinase pathways by mimicking IGF-1 and binding to IGF-1 receptor to promote thyroid carcinogenesis (43). This also reflects that the presence of T2D is associated with insulin resistance, leptin resistance, increased oxidized low-density lipoprotein cholesterol, and obesity, which are present, but to a lesser extent, in T1D. Furthermore, the majority of tumors occurred in patients with T2D, postulating that cancer mainly exists in the older people, in which T2D is more frequent (44). Therefore, an increase in thyroid cancer incidence in T1D may be an artefact of diagnosis because clinicians may pay more medical attention to these patients than non-DM individuals (29). In addition, exogenous insulin is an absolute demand for patients with T1D because they lack endogenous insulin. Moreover, unlike patients with T2D, patients with T1D lack a long prediabetes and diabetes history with compensatory endogenous hyperinsulinemia. As hyperinsulinemia decreases IGF-binding protein–1 (IGFBP-1) and IGFBP-2 levels, insulin may reduce certain IGFBP levels and increase bioavailable IGF levels to indirectly enhance the IGF–IGF-1R signaling pathway. In the situation of diabetes, hyperinsulinemia may therefore directly enhance tumor growth and progression or indirectly promote malignant transformation through IGF-1 signaling (45). Second, the increase of TSH levels is three times more common in patients with D2M than in those without DM (46). Chronic higher level of serum TSH was related to an elevated likelihood of differentiated thyroid cancer and more aggressive tumor stage (47). Interestingly, patients with malignancy may have normal TSH levels, whereas patients with benign tumors often have low TSH levels caused by toxic nodules, goiter, and Graves’ disease. Thyroid hormones have been implicated in promoting thyroid inflammation and thyroid cancer through genomic and non-genomic (membrane integrin receptor) actions. Genomic action of thyroid hormones promotes thyroid carcinogenesis by binding to specific nuclear receptors, but accumulated evidence suggests that the activation of the MAPK signaling pathway is the pathophysiological mechanism, which has been noted in the pathogenesis of papillary thyroid carcinoma. Recently, a novel pathway mediated by a membrane receptor located in integrin αVβ3 has been revealed. The proliferative and angiogenic effects of THs have been postulated through this mechanism. It is not yet clear whether the tumorigenic action noted in other neoplasms may play a role on DTC (48). Third, increased oxidative stress caused by hyperglycemia influences tumor cell growth and proliferation (17). Patients with DM suffered from increased permanent pro-inflammatory and oxidative stress caused by metabolic abnormalities. The intracellular anti-oxidant capacity reduced by prolonged inflammatory responses may increase the risk of carcinogenesis of susceptible cells (49). Increased oxidative stress determines thyroid cell inflammatory effects through Toll-like receptor (TLR) activation. Increased TLR expression, activation, and signaling adaptor molecules were found in peripheral blood mononuclear cells from patients with autoimmune thyroid disease (AITD), and TLRs may participate in the pathogenesis of AITD. A significant elevation in TLR endogenous ligands was also observed in the serum of AITD group (50). Moreover, accumulating evidence indicates the association between overactivation of TLR signaling and thyroid cancer progression (51, 52). This reveals the molecular mechanism of thyroid carcinogenesis caused by hyperglycemia to a certain extent. Finally, 70% of patients with DM have vitamin D deficiency (53). Inactivation of deiodinase II (DIO2) enzyme by a Vitamin D deficient environment in patients with DM leads to decreased glucose transporter 4 (GLUT4) transcription by skeletal muscle and adipose tissue, thus resulting in insulin resistance and thyroid carcinogenesis (34, 53).

The strengths of this study should be highlighted as follows: the analyses were limited to cohort studies, which could minimize the influence of biases such as recall and selection biases; the large sample size provided powerful statistical power for quantitative analysis of this correlation between DM and thyroid cancer risk, obtaining more robust results than any single study; subgroup analyses by type of DM and sex were performed to explore the associations between various kinds of DM and thyroid cancer risk and further investigate sex-specific thyroid cancer risk in any type of DM.

This meta-analysis also had some potential limitations. Because DM is shown in the vast majority of studies as a no or yes variable, it is not possible to assess the severity of DM and further explore its association with thyroid cancer risk. Moreover, we could not distinguish controlled vs. uncontrolled DM, because information on DM treatment was not available. In some studies, potential confounding factors were not adjusted such as obesity and age. Although they were adjusted, the adjustment models varied across the selected studies, which might affect the validity of the results. TSH levels and/or TNM classification or staging of thyroid cancer were not monitored in the original literatures included in this study, we cannot assess whether the difference of TSH levels exists between patients with DM and non-DM counterparts and also cannot explore the correlation between DM and progression of thyroid cancer. We expect this problem to be solved in the prospective studies in the future. Diabetes status was ascertained by a self-report in some previous studies. Thus, prediabetes and T2D can be misleading and undiagnosed glucose disorders account for 45.8% on a global scale. However, because of wide area coverage (nine countries), the large sample size (greater than 300,000, and a total of 11,091 cases of thyroid cancer), and the recent literatures accounting for the majority, the impact of this problem is minimized. We believe that our conclusion is still valid.

## Conclusion

Our results provided evidence that T2D could increase thyroid cancer risk in both sexes, underlining the demand for developing management and prevention strategies to mitigate thyroid cancer risk in any individual with T2D. Patients with GD are also at high risk for thyroid cancer. Further study is warranted to explore the association between T1D and thyroid cancer risk. Thus, given the increased thyroid cancer risk in patients with DM, DM prevention should be encouraged in all individuals, especially in the high-risk group.

## Data availability statement

The original contributions presented in the study are included in the article/**Supplementary Material**. Further inquiries can be directed to the corresponding author.

## Author contributions

HZ and W-WD conceptualized the research. W-WD and D-LZ conducted statistical analysis. Z-HW, C-ZL, and PZ contributed to data interpretation. W-WD wrote the manuscript draft. All authors contributed to the draft revision and approved the final draft of the manuscript.

## Conflict of interest

The authors declare that the research was conducted in the absence of any commercial or financial relationships that could be construed as a potential conflict of interest.

## Publisher’s note

All claims expressed in this article are solely those of the authors and do not necessarily represent those of their affiliated organizations, or those of the publisher, the editors and the reviewers. Any product that may be evaluated in this article, or claim that may be made by its manufacturer, is not guaranteed or endorsed by the publisher.
