# Supplementary material for: Different types of diabetes mellitus and risk of thyroid cancer: A meta-analysis of cohort studies
Source: Front Endocrinol (Lausanne). 2022 Sep 23;13:971213. doi: 10.3389/fendo.2022.971213 (PMC9537385; doi:10.3389/fendo.2022.971213)
Supplement: Supplementary file 1 [file Table_1.docx]

**The retrieval strategies and retrieval results of each database are shown in Tables 1-3**.

Table 1: PubMed

| No. | Content | Result |
| --- | --- | --- |
| #1 | Search: "Diabetes Mellitus"[Mesh] Sort by: Most Recent | 466,152 |
| #2 | Search: Diabetes[Title/Abstract] Sort by: Most Recent | 594,886 |
| #3 | Search: ("Diabetes Mellitus"[Mesh]) OR (Diabetes[Title/Abstract]) Sort by: Most Recent | 720,422 |
| #4 | Search: "Thyroid Neoplasms"[Mesh] Sort by: Most Recent | 57,080 |
| #5 | Search: (((Thyroid Neoplasm*[Title/Abstract]) OR (Thyroid Carcinoma*[Title/Abstract])) OR (Thyroid Cancer*[Title/Abstract])) OR (Thyroid Adenoma*[Title/Abstract]) Sort by: Most Recent | 46,995 |
| #6 | Search: ("Thyroid Neoplasms"[Mesh]) OR ((((Thyroid Neoplasm*[Title/Abstract]) OR (Thyroid Carcinoma*[Title/Abstract])) OR (Thyroid Cancer*[Title/Abstract])) OR (Thyroid Adenoma*[Title/Abstract])) Sort by: Most Recent | 69,315 |
| #7 | Search: (("Diabetes Mellitus"[Mesh]) OR (Diabetes[Title/Abstract])) AND (("Thyroid Neoplasms"[Mesh]) OR ((((Thyroid Neoplasm*[Title/Abstract]) OR (Thyroid Carcinoma*[Title/Abstract])) OR (Thyroid Cancer*[Title/Abstract])) OR (Thyroid Adenoma*[Title/Abstract]))) Sort by: Most Recent | 485 |

Table 2 Embase

| No. | Content | Result |
| --- | --- | --- |
| #1 | 'diabetes mellitus'/exp | 1,117,517 |
| #2 | diabetes:ti | 353,379 |
| #3 | #1 or #2 | 1,133,422 |
| #4 | 'thyroid tumor'/exp | 98,274 |
| #5 | 'thyroid neoplasm*':ab,ti OR 'thyroid carcinoma*':ab,ti OR 'thyroid cancer*':ab,ti OR 'thyroid adenoma*':ab,ti OR 'thyroid tumor*':ab,ti | 64,067 |
| #6 | #4 or #5 | 103,359 |
| #7 | #3 or #6 | 1,894 |

Table 3 Cochran Library

| No. | Content | Result |
| --- | --- | --- |
| #1 | MeSH descriptor: [Diabetes Mellitus] explode all trees | 34,070 |
| #2 | (Diabetes):ti,ab,kw | 89,556 |
| #3 | #1 or #2 | 92,177 |
| #4 | MeSH descriptor: [Thyroid Neoplasms] explode all trees | 674 |
| #5 | ("Thyroid Neoplasm"):ti,ab,kw OR ("Thyroid Carcinoma"):ti,ab,kw OR ("thyroid cancer"):ti,ab,kw OR ("Thyroid Adenoma"):ti,ab,kw OR ("Thyroid tumor"):ti,ab,kw | 1,530 |
| #6 | #4 or #5 | 1,644 |
| #7 | #3 and #6 | 65 |

Figure 1

Figure 2
